# Comparing Frequentist and Bayesian Methods for Factorial Invariance with Latent Distribution Heterogeneity

**DOI:** 10.3390/bs15040482

**Published:** 2025-04-07

**Authors:** Xinya Liang, Ji Li, Mauricio Garnier-Villarreal, Jihong Zhang

**Affiliations:** 1 Department of Counseling, Leadership, and Research Methods, University of Arkansas, Fayetteville, AR 72703, USA; jili@uark.edu (J.L.); jzhang@uark.edu (J.Z.); 2 Sociology Department, Vrije Universiteit Amsterdam, 1081 HV Amsterdam, The Netherlands; m.garniervillarreal@vu.nl

**Keywords:** factorial invariance, fit indices, model selection methods, maximum likelihood estimation, Bayesian estimation, measurement invariance, latent distribution heterogeneity

## Abstract

Factorial invariance is critical for ensuring consistent relationships between measured variables and latent constructs across groups or time, enabling valid comparisons in social science research. Detecting factorial invariance becomes challenging when varying degrees of heterogeneity are present in the distribution of latent factors. This simulation study examined how changes in latent means and variances between groups influence the detection of noninvariance, comparing Bayesian and maximum likelihood fit measures. The design factors included sample size, noninvariance levels, and latent factor distributions. Results indicated that differences in factor variance have a stronger impact on measurement invariance than differences in factor means, with heterogeneity in latent variances more strongly affecting scalar invariance testing than metric invariance testing. Among model selection methods, goodness-of-fit indices generally exhibited lower power compared to likelihood ratio tests (LRTs), information criteria (ICs; except BIC), and leave-one-out cross-validation (LOO), which achieved a good balance between false and true positive rates.

## 1. Introduction

Factorial invariance refers to the extent to which the relationships between the measured variables and the underlying latent constructs are equivalent across groups or time points (McDonald, 1989; Meredith, 1993; Millsap & Kwok, 2004). Factorial invariance is an important prerequisite for making meaningful comparisons of statistical properties across groups in social science research that employs factor analysis models. A prevalent approach to assess the factorial invariance is the multigroup confirmatory factor analysis (CFA; Jöreskog, 1971), which involves performing a series of increasingly restrictive invariance models, either in a forward or backward approach. Once factorial invariance is established, researchers can confidently assess the latent factor means and variances across different groups or time points.

Various factors such as sample size, data type and distribution, and model complexity can impact the detection of noninvariance (Cao & Liang, 2022; Sass et al., 2014; Yoon & Lai, 2018). One issue that has received little attention is the distribution of latent factor scores, including the latent factor mean and variance (Borsboom et al., 2008). Past studies have generally relied on the assumption of equal distributions of latent factors across groups. While a few studies have explored the variation in latent distributions between groups, the differences in latent means and variances have typically been small (Liang & Luo, 2020). In practice, however, unequal latent group distributions are commonly encountered, for example, when comparing cognitive constructs between gifted and general students, between persons with disabilities, and the general population. Detecting factorial invariance becomes more challenging when varying degrees of heterogeneity are present in the distribution of latent factors.

Factorial invariance testing can be conducted through multigroup CFA in a frequentist (e.g., Wu et al., 2007) or Bayesian framework (e.g., Shi et al., 2017). In the frequentist framework, maximum likelihood (ML) estimation is commonly used to derive model parameters by identifying a set of parameter values that maximize the likelihood of obtaining the observed data under the analysis model. In contrast, the Bayesian framework estimates parameters by updating the prior distributions with the data likelihood to generate posterior distributions. This updating process is typically implemented using Markov chain Monte Carlo (MCMC) algorithms, which iteratively sample from the posterior distribution.

In both frameworks, various fit measures are available to evaluate the comparative fit between two invariance models, though these measures are formulated differently in the frequentist and Bayesian contexts. In the frequentist approach, the computation of fit measures is typically based on point estimates (e.g., likelihood-based statistics). Goodness-of-fit indices, such as the chi-square test, root mean square error of approximation (RMSEA), comparative fit index (CFI), Tucker–Lewis index (TLI), Gamma hat (GH; Steiger, 1989), and McDonald fit index (MFI; McDonald, 1989), provide a quantitative measure of how well the model fits the data. The difference in their values between invariance models can be used to evaluate the factorial invariance. Information criteria (ICs), such as the Akaike information criterion (AIC), Bayesian information criterion (BIC), and their variates, are often used in model selection. These criteria combine a measure of model deviance and a penalty for model complexity, thus balancing model fit and parsimony. This trade-off is designed to prevent overfitting and enhance the overall generalizability of the selected models.

In the Bayesian framework, recent advancements have introduced Bayesian analogs of traditional frequentist fit indices, including the Bayesian versions of CFI, TLI, RMSEA, GH, and MFI (Garnier-Villarreal & Jorgensen, 2020; Hoofs et al., 2018). These Bayesian fit indices leverage the entire posterior distribution to evaluate model discrepancy and complexity, employing computational formulas analogous to their frequentist counterparts. Specifically, these measures are calculated at each iteration of the MCMC process to generate a posterior distribution of the fit indices. This posterior distribution can then be summarized using central tendency and variability metrics. In addition, ICs are also available within Bayesian CFA, including BIC and the deviance information criterion (DIC; Spiegelhalter et al., 2002). While DIC uses the full posterior distribution for the penalty term, it only employs posterior point estimates for the deviance term. The widely available information criterion (WAIC; Watanabe, 2010) and leave-one-out cross-validation (LOO; Geisser & Eddy, 1979; Vehtari et al., 2017) serve as more robust fully Bayesian selection methods, where the deviance is represented using the log pointwise predictive density (lppd; Gelman et al., 2013) computed at each sample draw from the entire posterior distribution. These Bayesian selection methods sample from the posterior distribution encompassing the full parameter space and provide flexible approaches to select models with effective incorporation of prior information.

Prior studies on factorial invariance have typically followed either a frequentist or Bayesian framework and focused on comparing fit measures within each respective framework (Shi et al., 2017; Liang & Luo, 2020). Only a few studies have compared frequentist and Bayesian methods in assessing factorial invariance (Liang & Luo, 2020; Lu et al., 2017), though they did not include the recently developed Bayesian goodness-of-fit indices (Garnier-Villarreal & Jorgensen, 2020; Hoofs et al., 2018). Since many Bayesian fit measures are adaptations of their frequentist counterparts, we aim to compare corresponding ML and Bayesian versions of fit measures to understand how they perform across different estimation frameworks in factorial invariance testing. In addition, the impact of heterogeneous latent distributions on model fit indices has not received sufficient attention in the literature, despite its prevalence in empirical research.

Therefore, our study purpose is twofold: first, to compare Bayesian and ML fit measures in factorial invariance testing; and second, to investigate the impact of latent distribution heterogeneity on the sensitivity of these fit measures under various simulation conditions. This comprehensive comparison aims to provide insights into the effectiveness and reliability of both estimation frameworks in addressing complex data structures and ensuring measurement fairness.

### 1.1. Factorial Invariance

A general multigroup CFA can be expressed as follows:

(1) $$y_{jg}=\nu_{g}+\Lambda_{g}\xi_{jg}+\delta_{jg},$$

where for subject *j* in group *g*, $y_{jg}$ is the *p* × 1 vector of observed scores (*p* is the number of items), $\xi_{jg}$ is *q* × 1 vector of latent factor scores (*q* is the number of factors) assuming $\xi_{jg}\sim MVN\left(\kappa_{g},\Phi_{g}\right)$ in which $\kappa_{g}$ is the latent mean vector and $\Phi_{g}$ is the covariance matrix of latent factors in group *g*, $\nu_{g}$ is a *p* × 1 vector of item intercepts in group *g*, $\Lambda_{g}\;$ is a *p* × *q* matrix of factor loadings in group *g*, and $\delta_{jg}$ is a *p* × 1 vector of error scores associated with person *j* in group *g*, following $\delta_{jg}\sim \mathrm{MV}N\left(0,\Psi_{g}\right)$ in which the error covariance matrix $\Psi_{g}$ is typically diagonal. The mean structure of the model is defined as follows:

(2) $$\mu_{y,\;g}=\nu_{g}+\Lambda_{g}\kappa_{g},$$

where $\mu_{y,\;g}$ is the mean vector of observed variables **y** in group *g*. The variance and covariance matrix Σ of the observed variables **y** in group *g* is delineated as follows:

(3) $$\Sigma_{y,g}=\Lambda_{g}\Phi_{g}\Lambda_{g}^{\prime }+\Psi_{g},$$

The common process of multigroup CFA for testing factorial invariance involves using a forward approach to compare the fit of a series of increasingly restrictive invariance models, including configural invariance (equal factor structure), metric invariance (equal factor loadings: $\Lambda_{g}=\Lambda$), scalar invariance (equal item intercepts: $\nu_{g}=\nu$ and $\Lambda_{g}=\Lambda$), and residual invariance (equal item residual variances: $\Theta_{g}\;$= Θ, $\Lambda_{g}=\Lambda$ and $\nu_{g}=\nu$) models (Millsap, 2011). The selection of the invariance model depends on comparing the fit of two models with different levels of parameter constraints. Factorial invariance is established if the two invariance models fit the data comparably. Otherwise, the less restrictive model is selected. In the present study, we focus on assessing the metric and scalar invariance testing because they are commonly regarded as adequate for cross-group of comparisons of latent means and variances and have received the most attention in methodological research (Cao & Liang, 2022; Liang & Luo, 2020; Little, 1997; Widaman & Reise, 1997).

### 1.2. Heterogeneous Latent Distribution

The process of measurement invariance seeks to separate two possible sources of differences. First, the true latent distribution differences, when the mean and variance of a latent variable differ between groups, and second, item differences unrelated to the latent factor, when a characteristic of the item relates to groups differences, also called item bias. An example of the first source of difference would be if we tested math ability between mathematicians majors and psychology majors in college, where we would expect different latent (math) levels. Meanwhile for the second source of difference, an example would be an analogy item mentioning Joe Montana, and then comparing men vs. women; more women might get the item wrong if they do not know who Joe Montana is, but this has nothing to do with their latent (verbal) ability. So, in the first example we have true group differences, while in the second one we have a biased item that can affect our interpretation of the latent differences.

From a more substantive point of reference, the expression of the psychological trait of extraversion could differ between individualistic cultures and collectivistic cultures, leading to differences in latent factor means and variances. Extraversion may be more valued and rewarded in individualistic cultures, resulting in a higher mean and variance in this group compared to collectivistic cultures, where social harmony and modesty may be more valued. This difference in latent distributions can have implications on the observed scores that are collected in practice. Consider a scenario in which the ratio of latent factor variance is $1:\phi_{g}$ and the ratio of latent factor mean is $1:\kappa_{g}$. Suppose latent factor distribution for group 1 is $N\left(0,1\right)$ and accordingly for group 2 is $N\left(\kappa_{2},\phi_{2}\right)$. The variance of the *p*th indicator can be expressed as $V\left(y_{1}\right)=\lambda_{1}^{2}+\psi_{1}$ for group 1 and $V\left(y_{2}\right)=\lambda_{1}^{2}\phi_{2}+\psi_{2}$ for group 2. The mean of the *p*th indicator can be expressed as $E\left(y_{1}\right)=\nu_{1}$ for group 1 and $E\left(y_{1}\right)=\nu_{2}+\lambda_{2}\kappa_{2}$ for group 2. An increase in latent mean and variance can lead to a corresponding increase in the mean and variance of the observed items, assuming items measure the same construct in both groups. The effect size for the latent mean difference between groups becomes smaller as the latent variance in group 2 increases: $\frac{\kappa_{2}}{\sqrt{\frac{1+\phi_{2}}{2}\;}}$. The increasing heterogeneity in latent factor distributions has a direct impact on the measurement of observed items.

In the least restrictive configural model, all model parameters including loadings and intercepts are freely estimated in every group *g*. For model identification purpose, latent factor distribution is typically constrained with mean zero and variance one by standardizing latent factor scores as $\eta_{g}^{\left(con\right)}=\frac{\eta_{g}-\kappa_{g}}{sqrt\left(\phi_{g}\right)}$. Accordingly, for group *g* with a latent distribution $N\left(\kappa_{g},\phi_{g}\right)$, the factor loadings can be re-expressed as $\lambda_{g}^{\left(con\right)}=\lambda_{g}^{2}\phi_{g}$ and the item intercept is $\nu_{g}^{\left(con\right)}=\nu_{g}+\lambda_{g}\kappa_{g}$ in the configural model. If the latent variance $\phi_{g}\;$ is high, it can result in large unstandardized loading estimates. Similarly, a higher latent mean $\kappa_{g}$ usually leads to a larger intercept in the configural model. In the metric invariance model, factor loadings are constrained equally across the groups even when noninvariant items are present. Typically, one group’s latent distribution is standardized, while the other group’s latent distribution is freely estimated. Misfit resulting from constraining factor loadings is then transformed into the estimation of latent factor variances as well as model fit evaluation. The scalar invariance model builds upon the metric invariance model by additionally constraining equivalent intercepts across groups, further translating misfit to the mean structure.

By constraining the item parameters (factor loadings and intercept) the model states that all differences are due to “true” latent factor differences. So, if an item is biased the measurement invariance constraints will incorrectly assume this bias does not exist, implying that the latent factor differences are biased now. Measurement invariance properly applied should separate true factor differences from item differences unrelated to the latent factor (bias).

The presence of heterogeneity in the latent distribution adds complexity to the test of measurement invariance and affects fit measures used to detect measurement noninvariance to varying degrees. Testing measurement invariance involves comparing fit indices obtained from two invariance models. The impact of heterogeneity on fit indices can vary, and it is crucial to explore how different measures of fit are influenced by the complexity introduced by heterogeneous latent distributions. The sensitivity of fit indices to the heterogeneity in latent distributions as well as the type and magnitude of noninvariance is not yet fully understood and warrants comprehensive investigation.

### 1.3. Frequentist Fit Measures

#### 1.3.1. Likelihood Ratio Test

The likelihood ratio test (LRT) is a chi-square based statistical test used in factorial invariance testing to compare the fit of two nested models. Define the log-likelihood, known as the deviance, as follows:

(4) $${\hat{D}}_{\mathrm{ml}}=-2\log p\left(y|{\hat{\theta }}_{\mathrm{ml}}\right),$$

where $p\left(y|{\hat{\theta }}_{\mathrm{ml}}\right)$ is the data likelihood given the ML estimates ${\hat{\theta }}_{\mathrm{ml}}$. When comparing two invariance models, the resulting difference between the two log-likelihood values ($\Delta {\hat{D}}_{ml}$) conforms to a chi-square distribution, where the degrees of freedom (*df*) are equal to the difference in the number of parameters between the two models. A statistically significant LRT indicates that the less constrained model fits better (e.g., configural over metric) and the model is less invariant across the groups. As sample size increases, even minor parameter differences may be regarded as significant by the LRT (Brannick, 1995; Cheung & Rensvold, 2002), and hence, multiple fit measures are developed to better control the rate of false positives.

#### 1.3.2. Goodness-of-Fit Index

Goodness-of-fit indices evaluate how well a model fits to observed data and most were developed based on the *T* statistic, expressed as follows:

(5) $$T_{ML}=\left(n-1\right)F_{ML}\left(\theta \right),$$

where *n* is the sample size, θ is the parameter vector, and the ML fit function, $F_{ML}\left(\theta \right)=\log\left|\Sigma \left(\theta \right)\right|+tr\left(S\Sigma^{-1}\left(\theta \right)\right)-\log\left|S\right|-p$, considers the model-implied covariance matrix $\Sigma \left(\theta \right)$, sample covariance matrix *S*, and the number of model parameters *p*. Goodness-of-fit indices fall into two categories: incremental fit indices, which measure improvement over a baseline model assuming no covariances, and absolute fit indices, which assess how well the model fits the data without referencing a baseline. Given $T_{T}$ and $T_{B}$ are the fit statistics for the target and baseline models, and $df_{T}$ and $df_{T}$ are their respective degrees of freedom, popular incremental fit indices include the comparative fit index (CFI; Bentler, 1990):

(6) $$CFI=1-\frac{\max\left[\left(T_{T}-df_{T}\right),0\right]}{\max\left[\left(T_{T}-df_{T}\right),\left(T_{B}-df_{B}\right),0\right]},$$

and Tucker–Lewis index (TLI; Tucker & Lewis, 1973):

(7) $$TLI=\frac{\left(T_{B}/df_{B}\right)-\left(T_{T}/df_{T}\right)}{\left(T_{B}/df_{B}-1\right)}.$$

Common absolute fit indices include the root mean square error of approximation (RMSEA; Steiger & Lind, 1980):

(8) $$RMSEA=\sqrt{\max\left(\frac{\left[\frac{T_{T}-df_{T}}{n-1},0\right]}{df_{T}}\right)}\;,$$

Gamma hat (GH; Steiger, 1989):

(9) $$Gamma\;Hat=p/[p+2\left(\frac{T_{T}-df_{T}}{n-1}\right)],$$

and McDonald fit index (MFI; McDonald, 1989):

(10) $$MFI=e^{-\frac{1}{2}\left(\frac{T_{T}-df_{T}}{n-1}\right)}.$$

Although CFI, TLI, and RMSEA are commonly reported, we also included MFI and GH in our investigation as they have been recommended for the test of measurement invariance (Cheung & Rensvold, 2002; Meade et al., 2008).

#### 1.3.3. Information Criterion

Information criteria are used for model selection and defined as a function of the deviance in (4) plus a penalty represented by the number of parameters.

(11) $$\mathrm{AIC}={\hat{D}}_{\mathrm{ml}}+2k,$$

(12) $$\mathrm{BIC}={\hat{D}}_{\mathrm{ml}}+k*\ln\left(n\right),$$

and

(13) $$\mathrm{SaBIC}={\hat{D}}_{\mathrm{ml}}+k*\ln\left(\frac{n+2}{24}\right),$$

where *k* is the number of model parameters. Lower values of ICs indicate better model fit. These ML-based ICs rely on the ML point estimate and thus their variability and distribution are difficult to quantify (Lu et al., 2016). Among the ICs above, BIC imposes the most severe penalty and tends to select a simpler model (Vrieze, 2012). AIC usually yields power close to the LRT for factorial invariance. The SaBIC adjusts the penalty based on the sample size. The AIC and SaBIC have been shown to perform relatively well in invariance model selection (Burnham & Anderson, 2002; Cao & Liang, 2022; Liang & Luo, 2020).

### 1.4. Bayesian Fit Measures

#### 1.4.1. Bayesian Fit Indices

Unlike frequentist estimation that relies on point estimates of the model’s deviance, Bayesian estimation computes the deviance and effective number of parameters at each MCMC iteration. These values are then substituted into the formulas for frequentist fit indices, enabling the computation of fit indices at each iteration. The fit indices from all iterations are aggregated, typically by averaging their posterior distributions, to provide overall measures of model fit. This approach incorporates parameter uncertainty, offering a more comprehensive evaluation of the model fit to the observed data.

Garnier-Villarreal and Jorgensen (2020) proposed an approach to adapt fit indices to Bayesian structural equation modeling (SEM). Specifically, at iteration *i*, the ML fit function *T* in the frequentist fit indices (Equations (6)–(10)) is replaced by $D_{i}^{obs}-pD$, where $D_{i}^{obs}$ is the discrepancy function based on the observed data, and pD represents the effective number of parameters, calculated as follows:

(14) $$pD=\bar{D}-D\left(\bar{\theta }\right)$$

Here, $\bar{D}=\frac{1}{I}\sum_{i=1}^{I}D\left(\theta_{i}\right)$ is the expected posterior deviance, and $D\left(\bar{\theta }\right)$ is the deviance at the posterior means of parameters $\bar{\theta }$. The *df* in frequentist fit indices is substituted by $p^{*}-pD$ to quantify model complexity, where $p^{*}$ denotes the unique sample moments. With these replacements, the distribution of realized values for various fit indices, including RMSEA, CFI, TLI, GH, and MFI, becomes available, which can be summarized using central tendency such as the mean (expected a posteriori; EAP), mode (modal a posteriori; MAP), or median, as well as percentile measures like the 2.5th and 97.5th percentiles to construct a 95% credible interval. For detailed formulas of each index, refer to Garnier-Villarreal and Jorgensen (2020). Research has shown that Bayesian fit indices yield similar results comparable to their frequentist counterparts with non-informative priors assigned (Edeh et al., 2025; Garnier-Villarreal & Jorgensen, 2020; Hoofs et al., 2018). Since these Bayesian fit indices are developed based on frequentist formulas, the same cutoff criteria are applied for comparing invariance models in this study.

#### 1.4.2. Bayesian Model Selection Methods

In the Bayesian LRT for measurement invariance testing, the deviance, $D\left(\theta_{i}\right)$, is computed at each iteration for the two invariance models, forming an empirical distribution of the LRT statistic that can be summarized by its central tendency. The difference in posterior mean deviances, $\Delta D\left({\bar{\theta }}_{EAP}\right)$, between the two models serves as a Bayesian analog to the frequentist LRT for assessing measurement invariance.

The DIC is often regarded as the Bayesian counterpart to the AIC. The DIC replaces the ${\hat{\theta }}_{\mathrm{ml}}$ in the deviance term of the AIC (Equation (11)) with posterior mean estimate ${\bar{\theta }}_{\mathrm{EAP}}$, and replaces the parameter count *k* with the effective number of parameters pD as follows:

(15) $$\mathrm{DIC}=D\left({\bar{\theta }}_{EAP}\right)+2pD.$$

The DIC is often described as partially Bayesian because the deviance term is computed using EAP point estimates, while the WAIC and LOO are viewed as fully Bayesian which capture uncertainty more comprehensively.

The WAIC is asymptotically equivalent to LOO, but computationally more efficient (Watanabe, 2010). Both make accurate predictions by utilizing information from the entire posterior distributions of model parameters while appropriately penalizing model complexity. WAIC is conceptualized as follows:

(16) $$\mathrm{WAIC}=-2\mathrm{lppd}+2p_{W},$$

where the log pointwise predictive density (lppd) approximates the deviance (Gelman et al., 2013) and $p_{W}$ indicates the effective number of parameters. The lppd is defined as follows:

(17) $$\hat{\mathrm{lppd}}=\overset{J}{\underset{j=1}{\sum }}\log\left[\frac{1}{I}\overset{I}{\underset{i=1}{\sum }}p\left(y_{j}|\theta^{\left(i\right)}\right)\right],$$

where $\theta^{\left(i\right)}$ indicates the parameter estimates at the *i*th iteration. The lppd first computes the expected pointwise predictive density (elpd) over *I* iterations for each subject *j*, and the log elpd is summed across all subjects to derive the lppd of the data. The approximation of lppd improves as the length of the MCMC chain becomes longer. The effective number of parameters $p_{W}$ is commonly estimated using the variance of the log pointwise predictive density over all subjects as follows:

(18) $${\hat{p}}_{W}=\overset{J}{\underset{j=1}{\sum }}{\mathrm{var}}_{\mathrm{post}}\left[\log p\left(y_{j}|\theta \right)\right].$$

This measure of ${\hat{p}}_{W}$ is most common and provides results closer to LOO (Gelman et al., 2014).

LOO estimates the expected log predictive density by leaving out one data point at a time:

(19) $$\mathrm{LOO}=-2{\mathrm{lppd}}_{\mathrm{LOO}},$$

where lppd_LOO_ is defined as

(20) $${\hat{\mathrm{lppd}}}_{\mathrm{LOO}}=\overset{J}{\underset{j=1}{\sum }}\log\left[\frac{1}{I}\overset{I}{\underset{i=1}{\sum }}p\left(y_{j}|\theta^{\left(j,i\right)}\;\right)\right].$$

Here, $\left(y_{j}|\theta^{\left(j,s\right)}\right)$ is the marginal likelihood from the *i*th iteration, excluding the *j*th subject.

The WAIC and LOO offer theoretical benefits over the DIC by leveraging full posterior distributions instead of point estimates. This extension allows the estimation of a measure of variability in the comparison between models (standard error of the difference), while methods based exclusively on the point estimate (like BIC, DIC) only report a difference without a reference of the variability. LOO excels with intricate data structures like network data and hierarchical data (Gelman et al., 2014). Additionally, accurate informative priors can enhance the sensitivity of the DIC, WAIC, and LOO in factorial invariance testing, particularly with small sample sizes (Liang & Luo, 2020).

## 2. Method

### 2.1. Design Factors

We investigated the impact of latent distribution heterogeneity on the invariance model selection using both ML and Bayesian fit measures. A one-factor CFA model with 12 items was employed. Two groups were considered, each with equal group sizes of 100, 300, and 600, resulting in total sample sizes of 200, 600, and 1200, typically encountered in both empirical and simulation studies (Cao & Liang, 2022; Sass et al., 2014). All factor loadings were all fixed at 0.7. The error variance was set to $\left(1-\lambda^{2}\right)*\phi_{g}$, where $\phi_{g}$ represents the latent factor variance for group *g*. These settings assumed that as the latent variance increased, the item variance would also increase proportionally. The latent factors were first generated from a normal distribution with mean zero and latent variance $\phi_{g}$. The observed indicators were then generated according to Equation (1), also following a normal distribution.

#### 2.1.1. Latent Factor Distribution

In the reference group (group 1), the latent factor distribution was standardized with a mean of zero and a standard deviation of one. For the focal group (group 2), the latent factor means were varied at 0 and 0.8, and the latent variances were set to 1 (no difference), 2, and 4 times that of Group 1, resulting in small, medium, and large latent variance ratios of 1, 2, and 4, respectively.

#### 2.1.2. Noninvariant Data

To generate noninvariant data, we varied the proportion of non-invariant items, location of noninvariance, and magnitude of noninvariance. Specifically, the proportions of noninvariant items were set at 8.3% (1 item) and 33.3% (4 items). The noninvariance was introduced in the factor loadings and item intercepts. For magnitudes of noninvariance, we chose 0.15 and 0.25 for both factor loadings and item intercepts. In particular, for group 2, the factor loadings for the noninvariant items were reduced by 0.15 and 0.25 compared to group 1, resulting in factor loadings of 0.55 and 0.45, respectively. Similarly, item intercepts in group 2 were 0.15 and 0.25 lower than those in group 1, leading to intercepts of −0.15 and −0.25 for the two levels of noninvariance, respectively. These settings for generating noninvariant data align with both methodological and empirical research in the measurement testing literature (Cao & Liang, 2022; Chen, 2007; Liang & Luo, 2020; Meade et al., 2008; Stark et al., 2006). In sum, we included 144 data generation conditions for noninvariant data (3 sample sizes × 3 latent variance ratios × 2 latent mean differences × 2 proportions of noninvariant items × 2 magnitude of noninvariance × 2 noninvariance locations).

#### 2.1.3. Invariant Data

To generate invariant data, all model parameters were set equal across groups. The invariant data conditions were established by fully crossing the three design factors, three sample sizes (100, 300, 600), three latent variance ratios (1, 2, 4), and two latent mean differences (0, 0.8), resulting in a total of 18 distinct data generating conditions.

### 2.2. Data Analysis

For each condition, we generated and analyzed 200 datasets using R (version 4.4.1). These datasets underwent analysis through maximum likelihood estimation using the “lavaan” package (Rosseel, 2012), and through Bayesian estimation using the “blavaan” package (Merkle et al., 2021). The Bayesian implementation was through Stan (Stan Development Team, 2023). In Bayesian estimation, model parameters were assigned non-informative priors using the blavaan default settings, indicating minimal prior knowledge and allow the data to primarily inform the estimates. Specifically, factor loadings $\lambda \sim N\left(0,\;10\right)$, intercepts $\nu \sim N\left(0,\;32\right)$, factor variances ϕ~Gamma(1, 0.5), and error variances ψ~Gamma(1, 0.5), where the second hyperparameter in the priors indicates standard deviation. The Hamiltonian Monte Carlo (HMC; Betancourt & Girolami, 2015; Neal, 2011), a MCMC sampling method, was employed to efficiently explore the landscape of posterior distributions and improve convergence rates. We set the burn-in iterations to 5000 and drew 5000 samples from each of three chains, resulting in a total of 15,000 posterior samples for summarization. It is to note that calculating incremental fit indices requires fitting a baseline model with the same number of burn-ins and samples as the proposed model, which allows for computing the incremental fit at each iteration. Therefore, the burn-ins and sample sizes were kept fixed throughout this study. To assess convergence, the potential scale reduction factor (PSR; Gelman et al., 1996), also known as $\hat{R}$, and the effective sample sizes (ESS; Gelman et al., 2013) were used. A $\hat{R}$ value below 1.05 for all parameters indicates convergence (Gelman et al., 1996). We also monitored the ESS to assess the independence and quality of the MCMC samples.

Three analysis models, configural, metric, and scalar invariance models, were fitted. In the configural invariance model, factor means were set at 0 and factor variances at 1 for both groups. The metric model maintained the reference group’s factor variance at 1, while freely estimating the focal group’s factor variance, with factor means still fixed at zero for both groups. In the scalar model, the reference group’s factor mean and variance were fixed at 0 and 1, respectively, allowing both to vary freely in the focal group.

To analyze metric noninvariant data, we compared the fit of configural and metric invariance models, while for scalar noninvariant data, we compared metric and scalar models. True positive (TP) rates were calculated as the proportion of replications correctly selecting the less restrictive model (configural over metric for metric noninvariance, and metric over scalar for scalar noninvariance). For invariant data, configural, metric, and scalar models were fitted to each dataset. Metric invariance was assessed by comparing configural and metric models, and scalar invariance by comparing metric and scalar models. False positive (FP) rates were defined as the proportion of replications incorrectly favoring the less restrictive model in the absence of noninvariance.

### 2.3. Outcome Evaluation

This study evaluated the performance of LRT, CFI, TLI, RMSEA, MFI, and GH within both frequentist and Bayesian frameworks. In addition, ML-based criteria (AIC, BIC, SaBIC) and Bayesian model selection methods (DIC, BIC, WAIC, LOO) were included in the analysis. For comparing two invariance models, the more restricted model (e.g., metric over configural) was chosen if the LRT was not significant at the 0.05 alpha level, or if fit indices met thresholds: ΔCFI < 0.01, ΔTLI < 0.01, ΔRMSEA < 0.015, ΔMFI < 0.02, or ΔGH < 0.001 (Chen, 2007; Cheung & Rensvold, 2002). For model selection criteria AIC, BIC, SaBIC, DIC, WAIC, and LOO, the model with the smaller value was selected. Furthermore, a factorial ANOVA, including eta-squared calculations, was conducted to evaluate how much variance in model fit differences (e.g., ΔCFI between configural and metric models during metric invariance testing) could be explained by design factors.

## 3. Results

### 3.1. Model Convergence

All replications converged in both ML and Bayesian estimations across conditions. In Bayesian analyses, the maximum $\hat{R}$ across all parameters and replications was 1.004, and the minimum ESS was 2589 per MCMC chain. The average $\hat{R}\;$ was 1.0009 and the average ESS was 4760 per chain. These metrics indicate that the Bayesian solutions were well converged and supported by a sufficient number of effective sample sizes. Convergence diagnostic plots for sample parameters can be found in the Supplementary Materials. The computational time for Bayesian estimation using Stan varied across simulation conditions and samples. In the largest sample size conditions (600 per group) with most noninvariant items, each invariance model typically completed estimation within 5 min. Computing Bayesian ICs took approximately 3 min, and computing Bayesian fit indices (e.g., BCFI, BRMSEA) took around 6 min. In conditions with smaller sample sizes, the computational time was correspondingly reduced. The above time estimates are based on a single dataset. The simulation was run using parallel function within Stan as well as parallel execution across multiple replications to maximize efficiency on a server with dual Intel Xeon Gold 5420+ processers and 256 GB of RAM.

### 3.2. Anlaysis of Variance

To gain a deeper understanding of the factors influencing the discrepancy in fit measure values between the two invariance models, a full factorial analysis of variance (ANOVA) was performed. The ANOVA examined the differences in fit values between the two invariance models, taking into account design factors including latent mean difference, latent variance ratio, number of noninvariant items, magnitude of noninvariance, sample size, and their interactions up to the fifth degree. Table 1 presents the ANOVA results, including main effects and second degree interaction effects that exhibit a moderate effect size based on eta-squared ($\eta^{2}$) greater than 0.0588 (Cohen, 1988). The $\eta^{2}$ indicates the percentage of variances in fit value differences that can be attributed to each design factor and their interactions.

In metric invariance testing, variations in latent means and latent variances did not contribute to the differences in fit values between the configural and metric models. The number and magnitude of noninvariant items accounted for 11% to 55% of the variances across all fit measures. The sample size had a substantial impact, explaining 43–49% for the LRT (ML, Bayes), AIC, DIC, WAIC, and LOO, and 9–17% for the BIC and RMSEA, while the contribution was negligible for the CFI, TLI, MFI, and GH. For all fit indices, the BIC and SaBIC, the number and magnitude of noninvariance interacted, with $\eta^{2}$ ranging from 6% to 26%. These factors also showed some interaction with sample size, particularly for the BIC and RMSEA. Similar patterns emerged in scalar invariance testing regarding the main effects of the number and magnitude of noninvariance and sample size. However, a key difference was that latent variance ratios explained about 9–15% of the variances across all fit measures, in contrast to the minimal percentage observed in metric invariance testing. Latent variance ratios also interacted with the number and magnitude of invariance (CFI, TLI, MFI) as well as sample sizes (BIC). Compared to their ML counterparts, the number of invariant items had a smaller impact on Bayes MFI and Bayes RMSEA but a greater effect on Bayes TLI and Bayes GH, with the loading differences showing the most pronounced effects.

### 3.3. False Positive Rate

**Figure 1:**
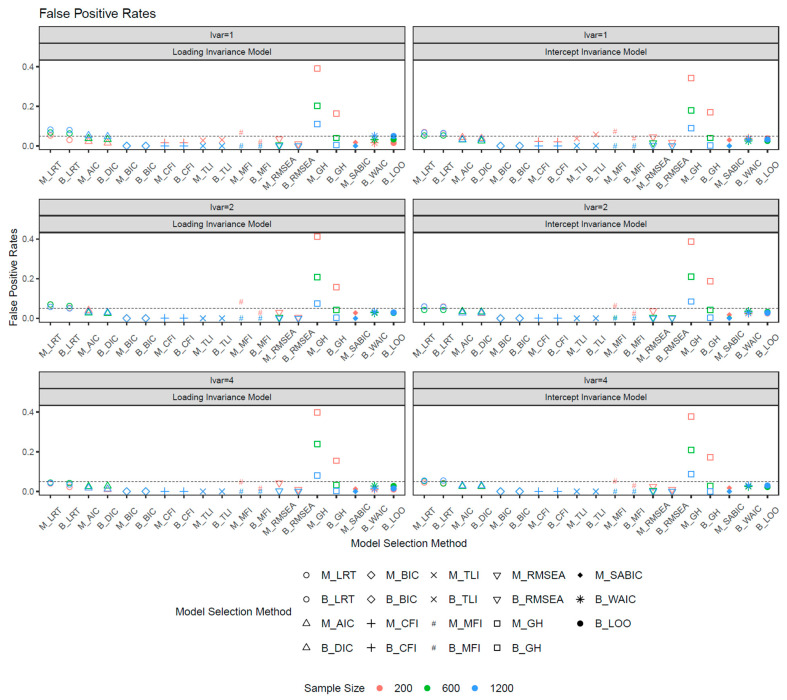
False positive rates. *Note*: lvar = latent variance ratio; M_ = ML selection method; B_ = Bayesian selection method.

Since the latent mean difference did not influence the fit measures from the ANOVA analysis, the reported results below combined data from both latent mean differences of 0 and 0.8. Figure 1 shows the false positive rates for both loading and intercept invariance models. Assuming a binomial distribution, the observed rates of FPs within the 95% confidence interval of [0.02, 0.08] ($0.05\pm 1.96*\sqrt{0.05*\left(1-0.05\right)/200}$) can be considered acceptable. For loading invariance models, larger sample sizes were associated with higher FP rates in the CFI, TLI, MFI, RMSEA, GH, and SaBIC, but lower FP rates in the LRT, AIC, DIC, WAIC, and LOO. In intercept invariance models, smaller sample sizes resulted in similar or higher FP rates. Among all fit measures, the GH exhibited the highest FP rates, reaching up to 0.42 with a sample size of 200. The ML-based GH show notably higher FP rates than its Bayesian counterpart. While FP rates for Bayesian GH were controlled at sample sizes of 600 or more, ML-based GH remained slightly inflated even at a sample size of 1200. Both ML and the Bayesian LRT exhibited slight FP rate inflation, particularly with larger sample sizes and equal latent variances, whereas increased latent variance mitigated this inflation. The remaining fit measures effectively controlled the FP rates across both ML and Bayesian frameworks.

### 3.4. True Positive Rate

**Figure 2:**
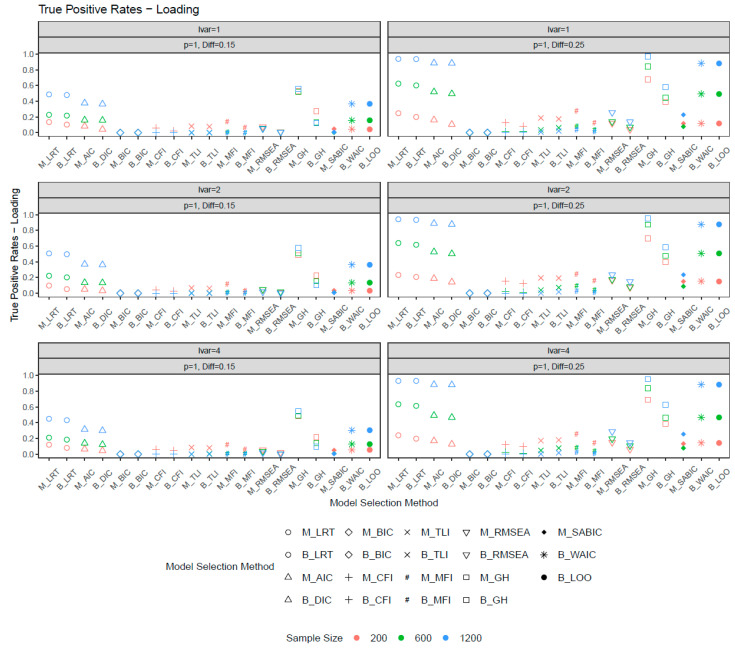
True positive rates for loadings when the number of noninvariant items equal to 1. *Note*: lvar = latent variance ratio; M_ = ML selection method; B_ = Bayesian selection method; *p* = number of noninvariant items; Diff = magnitude of noninvariance.

**Figure 3:**
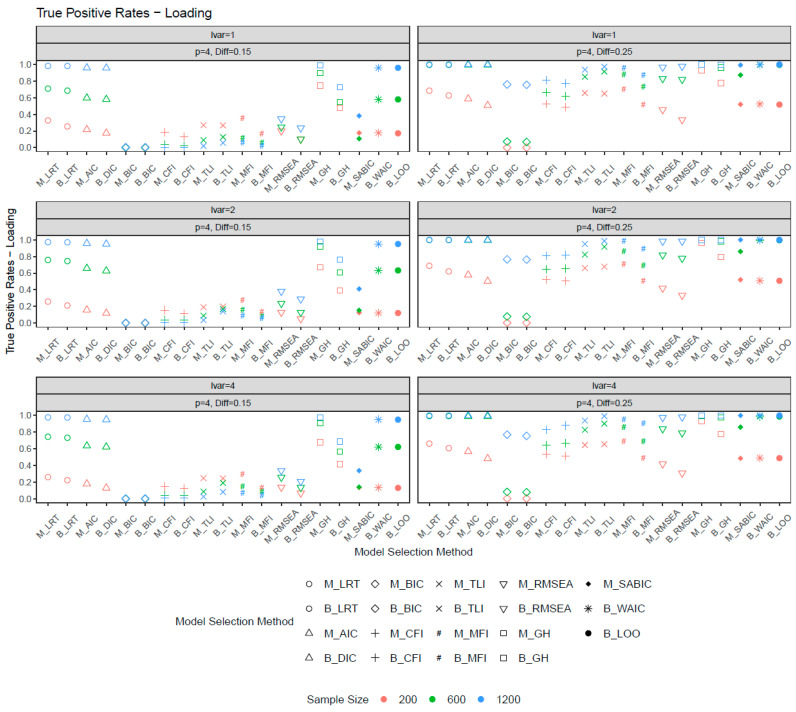
True positive rates for loadings when the number of noninvariant items equal to 4. *Note*: lvar = latent variance ratio; M_ = ML selection method; B_ = Bayesian selection method; *p* = number of noninvariant items; Diff = magnitude of noninvariance.

Figure 2 and Figure 3 show the true positive rates for the metric invariance models. In general, increasing the number of noninvariant items and noninvariance magnitude improved the true positive rates. The ML and Bayesian fit measures performed similarly in selecting invariance models, except for GH, where ML-based GH exhibited higher power than its Bayesian counterpart. Among all model selection methods, ML-based GH and both ML- and Bayesian-based LRT exhibited the highest true positive rates. However, ML-based GH also had inflated false positive rates, making it less ideal for model selection unless the sample size was sufficiently large. The LRT (ML, Bayes), AIC, DIC, WAIC, and LOO provided a good balance between false positive and true positive rates. Common fit measures such as the CFI, TLI, RMSEA, MFI, BIC, and SaBIC showed limited sensitivity in detecting metric noninvariance unless the sample size or noninvariance were sufficiently large. When the number of noninvariant items was four and the invariance difference was 0.25 (condition with the largest noninvariance), all fit measures demonstrated high power with a large sample size. For smaller sample sizes, some methods, such as the BIC and RMSEA, exhibited inferior performance. With relatively large noninvariance, greater sample sizes were associated with increased power. However, when the degree of noninvariance was modest, fit measures such as the CFI, TLI, and MFI demonstrated greater power with smaller sample sizes. Across the three latent variance ratios, sensitivity in selecting loading invariance models was largely unaffected by the latent variance ratio.

**Figure 4:**
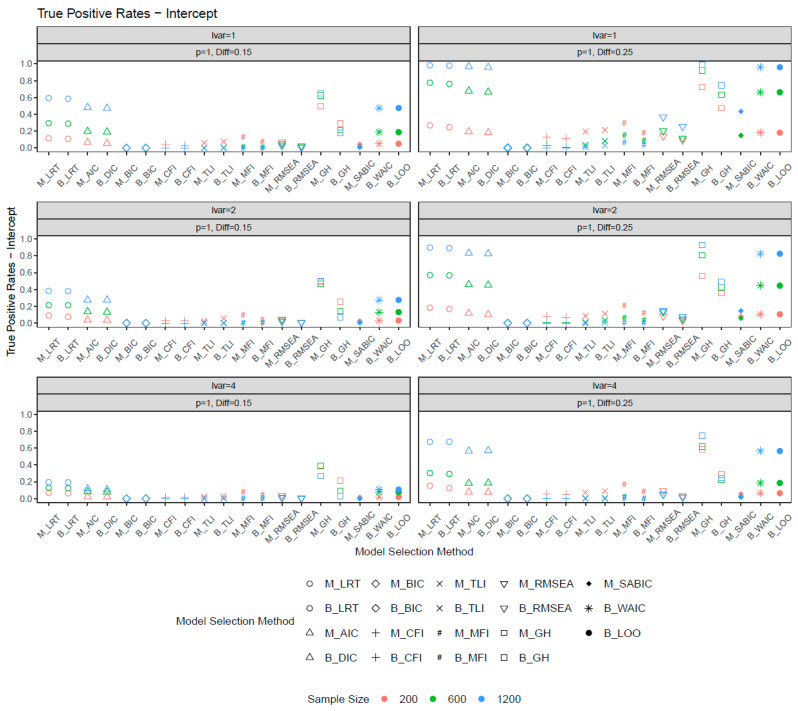
True positive rates for intercepts when the number of noninvariant items equal to 1. *Note*: lvar = latent variance ratio; M_ = ML selection method; B_ = Bayesian selection method; *p* = number of noninvariant items; Diff = magnitude of noninvariance.

**Figure 5:**
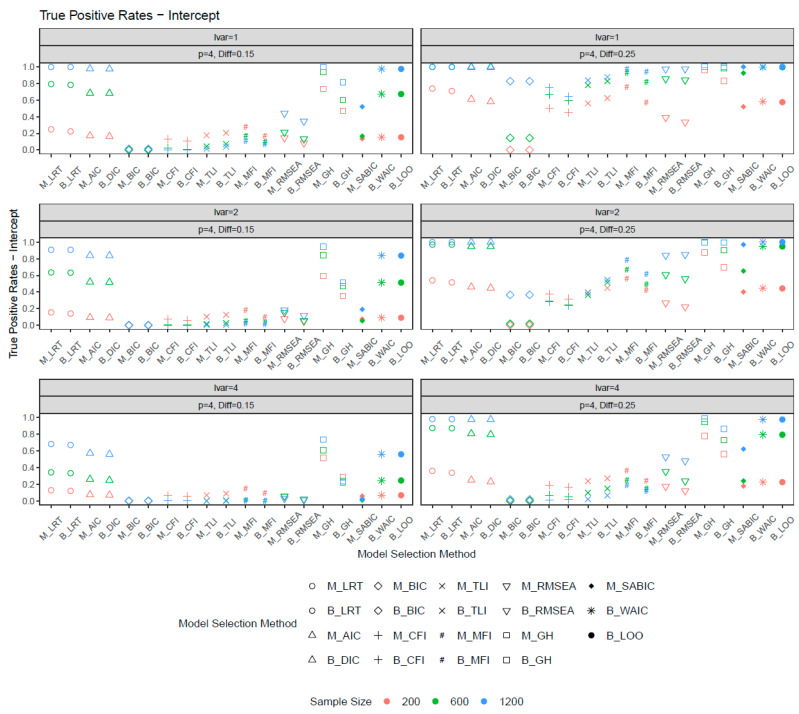
True positive rates for intercepts when the number of noninvariant items equal to 4. *Note*: lvar = latent variance ratio; M_ = ML selection method; B_ = Bayesian selection method; *p* = number of noninvariant items; Diff = magnitude of noninvariance.

Figure 4 and Figure 5 illustrate the true positive rates for the scalar invariance models. The overall pattern of TP rates was similar between the metric and scalar invariance models. However, the latent variance ratio had a greater impact on detecting scalar noninvariance than loading noninvariance. An increase in the latent variance ratio notably reduced the power of detecting intercept differences between groups. For large sample sizes (*n* = 1200) and an increased number of sizable noninvariant items, commonly used fit indices such as the CFI, TLI, RMSEA, and MFI achieved high power (>0.80) when the latent variance ratio was 1. However, as the latent variance ratio increased to 4, the power of the CFI, TLI, and MFI dropped dramatically, while RMSEA was slightly less sensitive to the difference in latent variances. This pattern was observed for both ML- and Bayesian-based fit indices. Notably, the Bayesian CFI, MFI, RMSEA, and GH had lower power than their ML counterparts, whereas the Bayesian TLI demonstrated higher power. For the CFI, TLI, and MFI, power decreased with increasing sample size. For other model selection methods, the LRT (ML, Bayes), AIC, DIC, WAIC, and LOO exhibited improved power as sample size and noninvariance magnitude increased. These methods also showed less sensitivity to latent variance heterogeneity and achieved the best balance between false positive (FP) and TP rates. Conversely, the BIC had the lowest power and was most affected by latent variance differences (e.g., *p* = 4, Diff = 0.25). Although the LRT demonstrated slightly better power than other selection methods, it should be noted that the LRT may slightly inflate FP rates.

## 4. Discussion

Heterogeneity in latent factor distributions is common when assessing factorial invariance within the CFA framework. This study examines how the change in latent means and variances between groups affects the sensitivity of model selection methods to detect noninvariance in factorial invariance testing across various conditions. By comparing frequentist and Bayesian model selection methods, it highlights their respective strengths and limitations in identifying heterogeneous latent structures. The findings enhance understanding of the complexities in assessing factorial invariance and offer guidance on selecting appropriate fit measures under such conditions.

The heterogeneity in latent variances between groups had a more pronounced effect on scalar invariance testing than on metric invariance testing across all fit measures. With a latent variance ratio of 1:1 and the latent factor in the reference group standardized, a 0.8 difference in latent means yielded an effect size of 0.8, considered large according to Cohen’s guidelines (Cohen, 1988). Conversely, with a latent variance ratio of 1:4, the effect size for a 0.8 mean difference was calculated as $d=\frac{\mu_{1}-\mu_{2}}{\begin{array}{c} \sqrt{\left(\frac{\sigma_{1}^{2}+\sigma_{2}^{2}}{2}\right)} \end{array}}=\frac{0.8}{\begin{array}{c} \sqrt{\left(\frac{1+4^{2}}{2}\right)} \end{array}}=0.27$, indicating a small to moderate effect size. Larger latent variances lead to greater variability in observed scores while smaller effect sizes between latent means, potentially diminishing the power to detect differences in group intercepts. In contrast, factor loadings—analogous to slopes in regression analyses—remain comparatively less affected. Latent mean difference, on the other hand, did not influence the selection of invariance models.

Moreover, we believe that the factor variance has a stringer effect on measurement invariance than the factor means due to the structure of the $\chi^{2}$ statistic. As it is the distance between the observed covariance matrix and the model implied covariance matrix (Bollen, 1989; Kline, 2016). Where the factor variances have an effect in reproducing a large number of estimates of the implied covariance matrix, while the factor means have a role in the reproducing the model implied means, which are included in the $\chi^{2}$ calculations as a difference between the observed means. For this reason, and the results in the present simulation, latent variance differences could have a stronger impact in measurement invariance when it is tested with fit indices based on the $\chi^{2}$ test, as shown in the following implementation of the maximum likelihood discrepancy function:

(21) $$F_{\mathrm{ML}}=\log\left|\hat{\Sigma }\right|-\log\left|S\right|+trace\left(S\hat{\Sigma^{-1}}\right)-p+{\left(\bar{x}-\hat{\mu }\right)}^{T}\hat{\Sigma^{-1}}\left(\bar{x}-\hat{\mu }\right),$$

which compares the sample covariance matrix S to the model-implied covariance matrix $\Sigma \left(\hat{\theta }\right)$ (or simply $\hat{\Sigma }$), where *p* is the number of variables in the model, $\bar{x}$ is the vector of sample means, and $\hat{\mu }$ is the vector of model-implied means. The corresponding statistic is calculated as $\chi_{\mathrm{ML}}^{2}=N\times F_{\mathrm{ML}}$.

In scalar invariance testing, when inspecting the individual model fitting, the fit values for metric models remained stable across various latent variances, whereas scalar models generally showed improved fit with higher latent variances. Consequently, as the latent variance ratio increased, the difference in fit values narrowed, reducing the power to detect intercept noninvariance. With a sufficiently large sample size and considerable noninvariance, all fit measures achieved approximately 0.80 power for a latent variance ratio of 1. Goodness-of-fit indices and the BIC experienced dramatic power reductions when the latent variance ratio increased to 4. In metric invariance testing, latent variances had minimal influence on the fit of both the configural and metric models, implying that the power to detect loading noninvariance was largely unaffected by the latent variance ratio.

As several Bayesian fit measures were developed using frequentist formulas while retaining Bayesian properties through the full posterior space, the overall performance of ML and Bayesian methods in selecting invariance models was comparable. Bayesian selection methods in general yielded slightly lower power than their ML counterparts, except for the TLI, where Bayesian methods demonstrated comparable or slightly higher power. In addition, an interaction between sample size and the degree of noninvariance was observed in the goodness-of-fit indices: smaller sample sizes led to higher power when noninvariance was small, but lower power when noninvariance was relatively large. Although this may seem counterintuitive, it partially aligns with the existing literature (Cao & Liang, 2022).

Among model selection methods, goodness-of-fit indices generally exhibited lower power to detect both metric and scalar noninvariance than the LRT, ICs (except the BIC), and LOO, which provided the best balance between false and true positive rates. The ML-based LRT and AIC performed similarly to the Bayesian-based DIC, WAIC, and LOO, likely due to the relatively simple one-factor structure and continuous normally distributed data, which allowed point estimates to effectively summarize the parameters and yield comparable power between ML and Bayesian methods. Under the current thresholds, most goodness-of-fit indices exhibited low power. However, GH, particularly within the ML framework, demonstrated good power, although it was associated with elevated false positive rates. In practice, optimal thresholds to balance Type I and Type II errors often depend on various factors such as sample size, model complexity, and the underlying distribution of the test statistic. These results suggest that adaptive thresholding approaches that are tailored to the specific context and methodological framework could be considered in future research.

### Implications and Future Research

The implications and recommendations from this study can be summarized below. First, for researchers seeking fit measures that balance false and true positive rates, the LRT (ML or Bayes), AIC, DIC, WAIC, and LOO are advisable. ML-based LRT and AIC are relatively time-efficient, while the Bayesian-based LRT, DIC, WAIC, and LOO require more computational time since they use all posterior samples. Nonetheless, the Bayesian approach offers greater flexibility through the incorporation of prior knowledge and uncertainty quantification in fit measures, warranting further research. When employing Bayesian fit measures, it is recommended to conduct a sensitivity analysis of priors in the context of factorial invariance testing (Depaoli & van de Schoot, 2017). Second, if the research objective is to detect noninvariance, GH may serve as a complementary measure to supplement the LRT, AIC, DIC, WAIC, and LOO due to its high power, although it may also yield inflated false positives using the conventional cutoff. Using multiple selection methods to determine the model invariance is advised. Third, unequal latent variances between groups have a minimal impact on metric invariance testing but can affect scalar invariance testing especially with high latent variance ratio for specific fit measures.

In practice, researchers should assess latent variances across configural, metric, and scalar invariance models. When substantial group differences in latent variances are detected (e.g., gifted verse general student populations), it is advisable to avoid relying on goodness-of-fit indices for scalar invariance testing and in cases where the number of noninvariant items in metric invariance testing is small, unless a sufficiently large sample size is available and a high degree of noninvariance is present. The choice between frequentist and Bayesian methods depends on factors such as the sample size, model complexity and data characteristics. If the assumptions for frequentist methods are generally met, frequentist selection methods are often preferred due to the computational efficiency. In situations where researchers have substantive prior knowledge they wish to incorporate or encounter issues such as convergence, small sample sizes, complex model structures or nonnormal data, Bayesian estimation may be more robust. Bayesian methods provide full posterior distributions of the fit measures, facilitating more informative assessment of model fit than point estimates from frequentist methods.

Further research could aim to characterize the distribution of fit indices from a Bayesian perspective and to investigate the impact of priors on model selection, guiding the development of optimal prior selection strategies that balance flexibility with robustness. Indices based on the chi-square discrepancy function are generally more sensitive to latent variance differences than latent mean differences, and it would need a specific simulation designed to test this. Additionally, expanding these investigations to encompass more complex models, such as those involving multiple latent factors or hierarchical structures, and diverse data types including nonnormal and ordered categorical data would provide valuable insights into the generalizability of the findings. In ML estimation, violation of normality assumption could affect the point estimates of fit measures to varying extent, while Bayesian approaches provide posterior distributions of fit indices that may be less sensitive to nonnormality, particularly when robust priors are employed. Both frameworks require careful sensitivity analyses to ensure that the invariance conclusions are not driven by distributional anomalies. Further, follow-up work could apply these methods to real datasets in educational or psychological settings where group comparisons are common to evaluate how well the current simulation results align with empirical findings.

## Figures and Tables

**Table 1:** Eta square from ANOVA of loading and intercept.

Fit Indices	Loading						Intercept									
	n.inv	diff	n	n.inv * diff	n.inv * n	diff * n	lvar	n.inv	diff	n	lvar * n.inv	lvar * diff	n.inv * diff	lvar * n	n.inv * n	diff * n
M_LRT	0.28	0.19	0.45				0.09	0.21	0.20	0.41						
B_LRT	0.26	0.18	0.49				0.09	0.20	0.19	0.43						
M_AIC	0.29	0.19	0.43				0.09	0.21	0.19	0.40						
B_DIC	0.26	0.17	0.48				0.09	0.21	0.19	0.41						
M_BIC	0.11	0.11	0.17	0.11	0.17	0.17		0.07	0.06	0.09			0.06	0.06	0.09	0.09
B_BIC	0.11	0.11	0.17	0.11	0.17	0.17		0.07	0.06	0.09			0.06	0.06	0.09	0.09
M_CFI	0.35	0.32		0.25			0.11	0.20	0.20		0.08	0.09	0.15			
B_CFI	0.35	0.32		0.26			0.10	0.18	0.18		0.08	0.08	0.14			
M_TLI	0.38	0.31		0.23			0.12	0.21	0.21		0.08	0.08	0.14			
B_TLI	0.43	0.30		0.21			0.11	0.23	0.23		0.06	0.07	0.15			
M_MFI	0.41	0.32		0.20			0.11	0.27	0.27				0.15			
B_MFI	0.36	0.32		0.23			0.11	0.24	0.24		0.07	0.07	0.16			
M_RMSEA	0.42	0.30	0.09	0.09			0.11	0.28	0.26	0.08			0.09			
B_RMSEA	0.36	0.27	0.09	0.14	0.06		0.09	0.26	0.23	0.08			0.12			
M_GH	0.41	0.32	0.13	0.06			0.15	0.31	0.30	0.11						
B_GH	0.55	0.34					0.15	0.36	0.34							
M_SABIC	0.43	0.30	0.07	0.12			0.11	0.28	0.25	0.07			0.10			
B_WAIC	0.27	0.18	0.47				0.09	0.21	0.19	0.41						
B_LOO	0.26	0.18	0.47				0.09	0.21	0.19	0.41						

lvar = latent variance ratio; n.inv = number of noninvariant items; diff = magnitude of noninvariance; *n* = sample size per group; n.inv * diff = interaction between the number of noninvariant items and magnitude of noninvariance; n.inv * n = interaction between the number of noninvariant items and sample size; diff * n = interaction between the magnitude of noninvariance and sample size; lvar * n.inv = interaction between the latent variance ratio and the number of noninvariant items; lvar * diff = interaction between the latent variance ratio and the magnitude of noninvariance; lvar * n = interaction between the latent variance ratio and the sample size.

## Data Availability

The simulated data will be available upon request.

## Supplementary Materials

The R code for data generation and analysis can be downloaded at https://osf.io/bekhd/?view_only=9c933d8085f0453cb1dd9caa6966a015, accessed on 25 March 2025.
